# Exercise Caution: Questions to Ask Adolescents Who May Exercise Too Hard

**DOI:** 10.3390/ijerph15040797

**Published:** 2018-04-19

**Authors:** Emma Forsén Mantilla, Johanna Levallius, Elin Monell, Andreas Birgegård

**Affiliations:** Centre for Psychiatry Research, Department of Clinical Neuroscience, Karolinska Institutet & Stockholm Health Care Services, Stockholm County Council, Norra Stationsgatan 69, 7 tr 113 64 Stockholm, Sweden; Johanna.levallius@ki.se (J.L.); elin.monell@ki.se (E.M.); Andreas.birgegard@ki.se (A.B.)

**Keywords:** compulsive exercise, healthy adolescents, eating disorder risk

## Abstract

When the primary goal of exercise is to compensate for food intake and to alter body shape and weight, it is considered compulsive and may be harmful. Compulsive exercise (CE) is important in the pathogenesis of eating disorders (EDs). Many healthy adolescents engage in CE too, and this may indicate a risk for EDs. Our aim was to learn more about ED risk factors tied to CE and to try to isolate questions to ask in order to probe for high ED risk in adolescents engaging in CE. Using two well-established instruments (the Structural Analysis of Social Behavior and the Eating Disorders Examination Questionnaire), we studied associations between ED variables and CE in healthy adolescent boys and girls. We examined gender-specific items to generate the best possible fit for each gender. Individuals with CE displayed significantly greater ED pathology and more self-criticism, and this pattern was stronger in girls than in boys. Risk factors for ED among individuals with CE differed slightly for boys and girls. We put forward a set of gender-specific questions that may be helpful when probing for ED risk among adolescents engaging in CE.

## 1. Introduction

Exercise and physical activity have many beneficial effects on both physical and psychological health [1]. Participation in organized sports has been linked to feeling more positive about one’s physical condition, higher self-esteem, a more positive body image, and lower levels of depression and anxiety [2,3]. For some, however, there is a darker side to exercise: when the primary goal for exercising is to alter weight or bodily appearance, to compensate for food intake, and/or to avoid guilt/negative affect if not exercising, exercise is considered compulsive and may cause both physical and psychological harm [4,5]. Compulsive exercise (CE) is a common symptom of eating disorders (EDs) in adolescents; 44% of girls and 38% of boys with ED reported CE in a large nationwide study of 13–17-year-old patients in specialized care in Sweden [6]. In adolescent ED patients, CE has been shown to maintain symptoms, increase the risk for relapse, and obstruct recovery [5,7,8]. CE is also prevalent in healthy boys and girls, and is associated with significant distress, more problematic eating, unrealistic expectations of thinness leading to life improvement, and higher levels of depression [9,10,11]. Problematic exercise has also been proposed to constitute a gateway to other forms of ED behaviors and cognitions [12] and has been linked to the development of EDs [7]. Thus, CE is potentially both an antecedent to and a symptom of ED pathology, and may be treated as an indicator of ED risk. However, not all who have subclinical symptoms of ED in adolescence develop the illness [13]. In this study, we aimed to investigate whether there are certain psychological variables linked to CE that signal added ED risk in a sample of healthy adolescents, and if those differ for boys and girls. From this, we aimed to devise a guide to probe for ED risk in adolescents who engage in CE.

EDs predominantly develop in early adolescence [14] and CE seems to be a risk behavior established at a relatively early age [9,10], making assessment of risk among young adolescents important but challenging, since many adolescents may not view their own ED-related behavior as compulsive or problematic [6,15,16]. Presence or absence of CE seems more important in relation to ED symptoms than amount of exercise or exercise frequency [17], i.e., when CE attitudes (such as exercising hard to alter body weight) are present, this contributes information about ED risk, regardless of exercise frequency. At present, the relationship between CE and other features of ED psychopathology remains somewhat unclear. The occurrence of CE in healthy adolescents may be partly explained by an investment in appearance, body dissatisfaction, and a drive for thinness [18,19]. Combined fasting and CE heightens distress and ED risk further [9,10]. However, boys and girls seem to differ slightly. A longitudinal study of risk factors for CE found a gender difference in that messages to become more muscular predicted CE in boys, whereas messages to be thin better predicted CE in girls [20]. They also found general ED psychopathology to be related to CE in girls, but not in boys.

Being highly self-critical, analogous to negative perfectionism, is associated with both CE and other ED pathology in adolescent boys and girls [18,21]. However, the association between self-criticism and ED pathology seems weaker in healthy boys compared to girls [21]. Although the overall pattern of predictors of CE looked similar for boys and girls, perfectionism imposed by others seemed to matter for CE in boys but not for girls [18].

Several validated measures exist to study CE, body dissatisfaction, fasting, and perfectionism/self-criticism [22,23,24]. However, administering these measures is time-consuming and interpreting the results requires prior knowledge of each instrument. In a primary care or school health service setting, there is a need for brief yet accurate questions to assess CE and risk of ED, and to decide whether further assessment is needed. The eating disorder examination questionnaire (EDE-Q: [25]) is the most well-known and widely used self-report measure of ED symptoms, measuring both underlying psychopathology and key behaviors such as CE. A recent factor analysis of the instrument [26] identified the items best capturing ED psychopathology in clinical girls, healthy girls, and healthy boys. Some items were central for all (e.g., desire to lose weight), while others were important in one group only. The factor structure looked different for healthy boys and girls (three factors vs. one), with much fewer items included in the boys’ model, suggesting, as have others, that the EDE-Q is better suited to girls [27]. Nevertheless, some items worked well for boys too. We aimed to identify effective EDE-Q items for each gender that relate to CE and provide information about their psychological importance. Further, self-criticism is not assessed in the EDE-Q, but is important for CE [20,28,29,30,31]. Hence, we examined the association between self-criticism and CE. The Structural Analysis of Social Behavior (SASB; [32]) measures self-criticism with four items, and they have been consistently related to ED pathology (and other forms of psychopathology) [6,21,33].

### The Present Study

Understanding CE and its associated features is important, as it may indicate risk for ED, but also because it has been associated with other forms of distress in healthy adolescents. In this study, we aimed to pinpoint self-critical and ED-associated attitudes that are related to CE in healthy adolescent boys and girls. The purpose was to isolate, based on the EDE-Q and SASB self-criticism, a set of questions that relate to CE and may signal risk for ED. This may yield a brief guide to aid those interviewing adolescents to uncover the psychological meaning of exercise behavior and whether risk is indicated. First, we examined differences in general ED pathology based on presence/absence of CE and gender. Second, we investigated the gender-relevant EDE-Q items reported in Forsén Mantilla et al. [26] and the four SASB self-criticism items in order to determine which items related most strongly to presence/absence of CE in boys and girls separately.

## 2. Methods

### 2.1. Participants

Adolescent boys and girls were recruited at schools in a Swedish Community. Out of a total of 675 adolescents in the age range 12–15 years who were eligible for the study, 171 students were absent (for unknown reasons) on the day of data collection and 22 students had incomplete forms, leaving 482 (71%) final participants. Out of these, 244 were boys and 238 were girls. Mean age, estimated based on which grade the participants were in, was 13.48 (*SD* = 0.50) for boys and 13.46 (*SD* = 0.50) for girls. Body Mass Index (BMI) was not recorded, but normal BMI for this age group in Sweden ranges between 17.6–18.9 for boys and 17.8–19.1 for girls [34].

### 2.2. Measures

*The Eating Disorder Examination Questionnaire 4.0 (EDE-Q* [25]*) adolescent version* [35] is a self-report questionnaire that measures ED pathology with 36 items, focusing on the past 14 days. Items focusing on cognitions and attitudes are rated on a 7-point scale (higher scores indicate more problematic attitudes: 0 = never and 6 = every day). The EDE-Q yields a global score and four subscales: Restraint, Eating Concern, Weight Concern, and Shape Concern, although the factor analytic structure of the measure typically does not reproduce those subscales (e.g., [26]). Items measuring occurrence and frequency of key ED behaviors such as CE are assessed by respondents specifying if they occur (yes/no) and if so, how many times during the past 14 days. Presence/absence of CE in this study was assessed with item 27 in the EDE-Q (“*over the past two weeks (14 days), have you exercised hard to control your shape or weight?”*). Frequency of CE is measured by item 28 in the EDE-Q (“*over the past two weeks (14 days), how often have you exercised hard to control your shape or weight?*”). This item was used for descriptive purposes to characterize the sample, but for the study aim we used item 27. The CE items in EDE-Q primarily apply to the dimension of CE concerning alteration of weight and/or bodily appearance. However, strong correlations between these and established CE measures (e.g., the Compulsive Exercise Test and the Exercise Beliefs Questionnaire) have been found [24], and item 27 has been successfully applied as a proxy for CE in two studies focusing on clinical populations [6,27], supporting its use as an approximation of CE in the present study. We used global EDE-Q score and the gender-specific items suggested in Forsén Mantilla et al. [26], when assessing ED pathology. The most salient items for boys and girls, respectively (see criteria under Statistical analysis below), were selected as independent variables for analysis of their association with CE. These items are summarized in short form in Table 1, and, of note, differ somewhat for boys and girls. The EDE-Q is common in research and clinical practice and has, aside from the factor structure, good psychometric properties, and both clinical and non-clinical reference data [21,35,36]. A Swedish translation of the instrument was used and the translation procedure has been described elsewhere [37].

*The Structural Analysis of Social Behavior (SASB)* is a 36-item self-report questionnaire that measures self-image in terms of eight clusters of self-directed behaviors, and Cluster 6 Self-blame, analogous to negative perfectionism and characterized by self-directed criticism, was used here. Items are rated on a 0 to 100 scale indicating increasing levels of agreement. The original version of the instrument has good psychometric properties [38,39]. Cronbach’s alpha for Self-blame in the Swedish version has been consistently high [21,40]. Self-blame is also the one self-image cluster most strongly associated with ED symptoms in healthy, symptomatic, and clinical individuals [21,41]. Cronbach’s alpha for Self-blame in this study was 0.80. The four Self-blame items that were examined regarding their relationship to CE are presented in Table 1.

### 2.3. Procedure

Parents and teachers received letters informing them about the aim and procedure of the study. Parents were informed about the study and encouraged to contact the project supervisor if they did not want their child to participate. No parent objected. Data were collected during school hours in the classrooms, over a two-week period. Final term MSc university students administered the questionnaires, adhering to a manual for administration. All participants were informed that participation was voluntary and their responses confidential. The student health care teams were informed, in case filling out the forms should cause worry or concern amongst the participants. The community Board of Education approved the study as well as the Ethical Review board (Dnr 2013/82-31/4).

### 2.4. Statistical Analysis

Sample characteristics, presence of CE, and ED risk scores (based on clinical significance cutoffs empirically derived based on [42]) are reported. Differences in Self-blame based on gender and presence/absence of CE were examined with independent samples *t* test. Effect sizes (Cohens *d*) were computed and considered small *d* ≥ 0.20, medium *d* ≥ 0.50, and large *d* ≥ 0.80.

Differences in ED pathology between individuals with and without CE were examined using both the standard global EDE-Q score (in the sample as a whole) and the gender-specific items suggested in Forsén Mantilla et al. [26], for boys and girls separately. A univariate analysis of variance was conducted with presence/absence of CE and gender as independent variables and global EDE-Q as dependent variable (for exploratory purposes, we also tested substituting the global EDE-Q score with a 7-item scale, as recently suggested by Machado and colleagues [43]). Thereafter, two one-way ANOVAs were conducted investigating presence/absence of CE in boys and girls separately, with each gender-specific scale as the dependent variables. Effect sizes are shown as Partial *η*^2^ and considered small *η*^2^ < 0.01, medium *η*^2^ < 0.06, and large *η*^2^ < 0.14.

For the second purpose of the study, all EDE-Q items with factor loadings above 0.70 for boys and/or girls separately in Forsén Mantilla et al. [26] were selected as independent variables and analyzed for their association with CE (Table 1). The four Self-blame items were used for both boys and girls. Due to the differences in scale ranges between the EDE-Q and SASB, all responses were standardized by *z*-transformation in boys and girls separately prior to analyses. In order to predict presence/absence of CE in boys and girls respectively, two Backward stepwise (Likelihood ratio) logistic regressions were conducted. This elimination procedure was used due to the exploratory nature of the study. It starts by including all variables in the model, thereafter excluding the variables least associated with the dependent variable, one at a time, until the best possible fit is achieved.

## 3. Results

### 3.1. Sample Characteristics

Presence of CE was found in 135 individuals (28%), and more girls (*n* = 83, 35%) than boys (*n* = 52, 21%) reported CE. Within the group reporting CE, girls reported a somewhat lower frequency than boys (*M* = 5.3, *SD* = 4.1; *M* = 5.9, *SD* = 5.4, respectively). Risk levels of ED pathology were found in 24% of the girls and 18% of the boys. Self-blame was significantly higher in girls (*M* = 29.1, *SD* = 24.1) compared to boys (*M* = 20.2, *SD* = 18.7; *t* = 4.53, *p* < 0.001; small effect: *d* = 0.42), and in individuals with CE (*M* = 33.6, *SD* = 24.1) compared to individuals without (*M* = 21.2, *SD* = 20.2; *t* = −5.70, *p* < 0.001; medium effect: *d* = 0.56).

### 3.2. CE and Overall ED Symptom Burden

Results indicated a significant main effect of CE, with individuals with CE displaying higher levels of ED symptoms as measured by the EDE-Q Global scale (*M* = 1.83, *SD* = 1.4; *M* = 0.66, *SD* = 0.90, respectively, *F*(1, 479) = 95.10, *p* < 0.001; *η*^2^*_partial_* = 0.166, a large effect) (note: substituting the Global EDE-Q with the 7-item version of the scale suggested by Machado et al. [41] produced similar results (CE: *M* = 1.86, *SD* = 1.4; Non-CE: *M* = 0.72, *SD* = 0.92, *F*(1) = 87.27, *p* < 0.001, *η*^2^*_partial_* = 0.154; girls: *M* = 1.44, *SD* = 1.34; boys: *M* = 0.64, *SD* = 0.86, *F*(1) = 54.84, *p* < 0.001, *η*^2^*_partial_* = 0.103; interaction: *F*(1) = 6.16, *p* < 0.05, *η*^2^*_partial_* = 0.013, with girls + CE: *M* = 2.25, *SD* = 1.37 and boys + CE: *M* = 1.21, *SD* = 1.18)). There was also a significant main effect of sex (*F*(1, 479) = 61.90, *p* < 0.001; *η*^2^*_partial_* = 0.114, a medium effect), with girls reporting more symptoms than boys (*M* = 1.41, *SD* = 1.35; *M* = 0.59, *SD* = 0.82, respectively). (Note: Two one-way ANOVAs comparing individuals with and without CE within each sex on the gender-specific total scale for girls and boys respectively (suggested in Forsén Mantilla et al. [25]) were conducted. Using these scales, the boys total score increased slightly and so did the score for boys with CE, but the overall pattern remained (results not shown)). The two main effects were qualified by a significant interaction effect (*F*(1, 479) = 9.21, *p* < 0.01; *η*^2^*_partial_* = 0.019, a small effect), with girls with CE reporting higher general ED pathology than boys with CE (*M* = 2.27, *SD* = 1.38; *M* = 1.14, *SD* = 1.14, respectively). When using the gender-specific EDE-Q scales as the dependent variables in two separate one-way ANOVAs, individuals with CE scored significantly higher (boys: *M* = 1.29, *SD* = 1.29, girls: *M* = 2.51, *SD* = 1.45) compared to individuals without CE (boys: *M* = 0.55, *SD* = 0.81, *F*(1) = 25.40, *p* < 0.001, and girls: *M* = 1.08, *SD* = 1.20, *F*(1) = 66.72, *p* < 0.001), with a greater effect size for girls than for boys *(η*^2^*_partial_* = 0.22 and *η*^2^*_partial_* = 0.09 respectively).

### 3.3. Associations between Individual Items and CE

#### 3.3.1. Girls 

The test of the full model against a constant only model was significant, indicating that the items as a set reliably distinguished between individuals with and without CE (*X*^2^ = 63.87, *p* < 0.001 with *df* = 3). The Likelihood ratio demonstrated that EDE-Q items 12 (Fear of weight gain) and 1 (Restraint over eating), and SASB item 35 (Self-doubt, putting self down), contributed significant shared variance (Table 2). Nagelkerke’s *R*^2^ of 0.33 indicated a moderate relationship. Classification success for these three items overall was 73.4%.

#### 3.3.2. Boys 

Like for the girls, a test of the full model against a constant model was significant (*X*^2^ = 18.80, *p* < 0.001 with *df* = 2). Two items contributed significantly (Table 2): EDE-Q item 32 (Dissatisfaction with weight) and EDE-Q item 2 (Dietary restraint), with a Nagelkerke’s *R*^2^ of 0.12. Classification success was 79.1%.

## 4. Discussion

**Figure 1:**
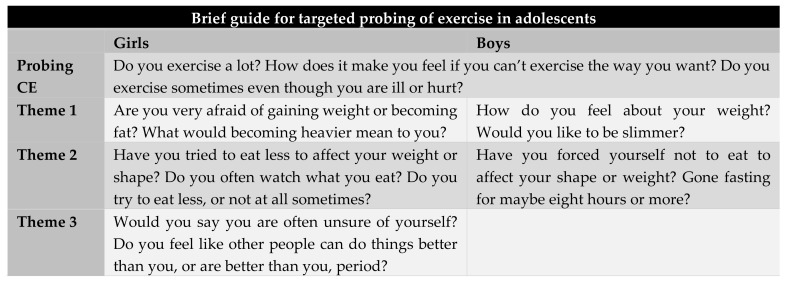
Brief guide for asking about eating disorder (ED)-related aspects in the presence of strenuous exercise, adapted from the items in the eating disorder examination questionnaire, and the structural analysis of social behavior that explained variance, in order of strength.

The purpose of the present study was threefold. First, we aimed to estimate the prevalence of CE (primarily with respect to altering weight and/or bodily appearance) in adolescents, where CE turned out to be common, with about one in five boys and one in three girls reporting CE. Second, we aimed to investigate the association of ED-related variables to CE, where we found that adolescents reporting CE had higher levels of ED pathology, scoring on average three times higher than non-CE adolescents. Third, we wanted to devise a brief guide for targeted probing of CE and ED by investigating how self-critical and ED-associated attitudes related to CE. Among boys, CE in combination with being dissatisfied with one’s body weight and restraining one’s diet indicated higher ED risk. Among girls, CE in combination with fear of gaining weight, restrictive eating, and self-doubt indicated higher ED risk. Below, we present a brief guide of relevant questions to ask healthy adolescents to assess the psychological importance of hard exercise with the goal of altering one’s body weight or shape and whether ED risk seems to be indicated (Figure 1). The “Probing CE” questions in the guide include central features of compulsivity: affective withdrawal symptoms when unable to exercise, and continuance (i.e., engaging in the behavior despite risks/negative consequences). These have been shown to be central in previous research [44] and are useful for querying about CE in clinical contexts.

A significant proportion of boys and girls reported the presence of CE (28%). CE was in turn related to greater levels of ED pathology and self-criticism. This pattern was stronger for girls, although significant for both genders. Not all individuals with subclinical symptoms develop EDs, yet this clearly signals a risk. Also, for both boys and girls, items signaling worries about body weight and dietary restraint were related to CE. This highlights how problematic exercise can be motivated by a wish to change one’s body shape and/or weight [4,5]. However, one interesting difference was that while fear of weight gain (item 12) seemed important in girls, weight dissatisfaction generally (item 32), which may also include shape/muscularity issues and dissatisfaction with slimness, was implicated as important in boys. This may suggest that questions to girls may be more specific and refer to fear of weight gain/becoming overweight, whereas boys may need to be queried more openly, with a wider range of possible body-changing motivations.

Together, the combination of symptoms suggested here may put the individual at risk for developing an ED. Numerous studies have established that maladaptive perfectionism predicts CE, ED, and psychopathology in general (e.g., [20,28,29,30,45,46]). A prominent feature of maladaptive perfectionism is excessive and negative self-evaluation [47]. In the current study, four self-criticism items were significantly correlated to CE. One of the items (self-doubt) remained in the final regression model for the girls but none remained in the final model for boys. This runs counter to the findings by Goodwin and colleagues [20], where self-oriented perfectionism and obsessive-compulsive traits predicted subsequent development of CE in boys, but not in girls. It is possible that their prospective and our concurrent data are responsible for this observed difference, as different variables may signal risk for future CE versus maintain it once present. As perfectionism plays such a prominent role in the development of various kinds of psychopathology, the role of negative self-evaluation in CE warrants further investigation.

Goodwin and colleagues [18] argue that sociocultural messages about body shape, weight, and exercise contribute to problematic and dangerous attitudes about exercise in adolescents, regardless of their weight status. With obesity and sedentary lifestyles increasing, encouraging exercise/physical activity in adolescence is important, but also requires care in order not to promote compulsive attitudes to exercise. Early detection of problematic behaviors is therefore important. Those who might detect problematic exercise or eating are most often not ED professionals, but school staff, coaches, and family members. Besides being attentive to CE attitudes and behaviors, they may need to enquire about dietary restraint, for instance, as signaled by our data. Based on our findings, we have devised a guide (Figure 1) that is intended for untrained adults close to an adolescent, not as a means to diagnose, but to provide them with informative questions to assess if there might be increased risk of CE and/or ED. This risk cannot be estimated by the sheer amount of exercise, as adolescents can exercise vigorously, and even be elite athletes, without developing CE or ED.

The prominent societal emphasis on physical appearance fosters a strong idealization of the thin and/or muscular body. Adolescents are especially susceptible to such ideals and pressures. A greater internalization of beauty ideals and appearance comparison with others early on in life seem associated with both exercise behavior and more eating psychopathology [48]. An emphasis on a thin ideal also contributes to stigma in overweight individuals, which increases risk of eating pathology [49]. Drawing on these studies and the current one, we believe health initiatives need to focus on encouraging fun, social, and cooperative attitudes toward sports and physical activity, where developing skills, rather than burning calories and achieving a certain body shape and/or weight, should be the prime goal.

### Strengths and Limitations

The present study has several strengths, including a large sample of both boys and girls who were systematically recruited in community schools. This implies good generalizability to children in similar ages. Further, the study uses validated and relevant measures, and examined age- and gender-specific items, which increased specificity. However, some limitations need to be considered. First, even though we used gender-specific items, the EDE-Q was primarily designed for females and it mainly corresponds to the thinness ideal. The athletic ideal might be equally problematic, particularly in males, where an emphasis on muscularity seems to be of importance, and such attitudes might not have been adequately addressed in this study. Second, CE was measured by only one item in the EDE-Q (i.e., presence/absence of hard exercise to control body weight/shape), and though there is good correspondence with other measures of CE [24], we do not capture all dimensions of CE with this measure. However, one aim of this study was to try to find brief yet accurate questions to assess CE and risk of ED in a primary care or school setting, for instance. As such, CE as measured with the EDE-Q nevertheless fills an important function, as an approximation of CE and a tool for deciding whether further assessment is needed. Another drawback was that we did not have data on participants’ BMIs, which is why possibly meaningful associations between BMI and CE could not be examined. Third, we have focused on presence/absence of CE rather than frequency in the present study, as presence/absence has been highlighted as the most important factor in relation to ED in women [17]. However, we cannot be certain that this also applies to males. Replicating the Mond and Calogero [17] study with male samples might therefore be important in the future. Fourth, we were unable to directly test whether presence of CE and high scores on any or all of the key ED aspects actually meant elevated risk of ED, as we lacked an objective measurement of such. ED risk can be assessed using the global EDE-Q [42], but since we used EDE-Q items as independent variables, it would be problematic to also assess clinical risk with the same measurement. Extending on our findings in future research, it would be interesting to see when ED risk (as measured by another objective instrument) is indicated in individuals with CE in combination with any or all of the ED aspects prominent here. Fifth, since the study is based on self-reports, symptoms and attitudes might be both over- and/or underreported. For instance, possible denial of CE and other symptoms is present among both adolescents with EDs and female athletes [6,50]. Interviews may be a good methodological addition in future research with similar populations. Also, due to both gender socialization and general cognitive development, girls in this age-span might have better introspective abilities and therefore provide more reliable self-ratings compared to boys. Sixth, since the study design was cross-sectional, no assumptions on causality can be made. Lastly, a larger proportion of students than expected was missing when data were collected. Since no attrition analyses could be conducted, generalizability might be decreased.

## 5. Conclusions

CE was highly prevalent in our sample of healthy adolescent boys and girls and related to increased ED-symptomatology and self-criticism. Based on established ED measures, a set of questions was isolated relating to CE that may signal compulsivity, risk for distress, and ED risk. The guide provided in this paper can aid in the assessment of the meaning of exercise, if CE and ED risk is indicated, and if further assessment might be warranted.

## Figures and Tables

**Table 1:** Overview of items tested for associations with CE in girls and boys.

Predictor Items	Girls	Boys
EDE-Q		
Item 1. Restraint over eating	X	
Item 2. Dietary restraint	X	X
Item 10. Flat stomach	X	
Item 11. Preoccupation with weight/shape	X	
Item 12. Fear of weight gain	X	
Item 13. Feelings of fatness	X	X
Item 14. Desire to lose weight	X	X
Item 15. Feelings of guilt after eating	X	
Item 29. Importance of weight	X	
Item 30. Importance of shape	X	
Item 32. Dissatisfaction with weight	X	X
Item 33. Dissatisfaction with shape	X	X
Item 35. Discomfort seeing body	X	X
Item 36. Discomfort exposing body	X	X
SASB		
Item 7. Self-accusation, blame and guilt, bad self	X	X
Item 24. Vengeful of self, self-punish	X	X
Item 25. Self-deception, forcefully diverting self	X	X
Item 35. Self-doubt, putting self down	X	X

Note: CE: compulsive exercise; EDE-Q: Eating Disorder Examination Questionnaire; SASB: Structural Analysis of Social Behavior.

**Table 2:** Logistic regression analysis of EDE-Q and SASB items in relation to compulsive exercise, with independent variables standardized to simplify interpretation due to the different scales in EDE-Q and SASB, listed in order of prediction strength. Coefficients and model tests from final stepwise model.

Predictor Variables	B	*S.E.*	Wald’s *Χ*^2^	*df*	*p*	Odds Ratio
Girls (*n* = 237)						
Constant	−0.76	0.16	21.44	1	<0.001	0.47
EDE-Q item 12. Fear of weight gain	0.72	0.19	14.07	1	<0.001	2.05
EDE-Q item 1. Restraint over eating	0.46	0.19	5.70	1	0.017	1.58
SASB item 35. Self-doubt, putting self down	0.36	0.17	4.76	1	0.029	1.43
Model test			*Χ* ^2^			
Overall model evaluation (Likelihood ratio test)			63.87	3	<0.001	
Boys (*n* = 246)						
Constant	−1.48	0.18	70.20	1	<0.001	0.23
EDE-Q item 32. Dissatisfaction with weight	0.47	0.14	10.53	1	0.001	1.60
EDE-Q item 2. Dietary restraint	0.37	0.13	7.65	1	0.006	1.45
Model test			*Χ* ^2^			
Overall model evaluation (Likelihood ratio test)			18.80	2	<0.001	

Note: EDE-Q: Eating Disorder Examination Questionnaire; SASB: Structural Analysis of Social Behavior.
